# P63 Parents report that healthcare professionals play an influential role in deciding whether to vaccinate against COVID-19: Results of a patient-led survey in Canada

**DOI:** 10.1093/rap/rkac067.063

**Published:** 2022-09-28

**Authors:** Jennifer Wilson, Wendy Costello, Saskya Angevare, Richard Beesley

**Affiliations:** Cassie And Friends Society, Vancouver, Canada; iCAN Ireland, Bansha, Ireland; European Network for Children with Arthritis, Geneva, Switzerland; KAISZ, Amsterdam, Netherlands; European Network for Children with Arthritis, Geneva, Switzerland; Juvenile Arthritis Research, Tonbridge, United Kingdom; European Network for Children with Arthritis, Geneva, Switzerland

## Abstract

**Introduction/Background:**

Vaccination of children and young people (CYP) with rheumatic and auto-inflammatory diseases is reported to be lower than amongst healthy peers. Whilst vaccination to confer protection against COVID-19 is now underway amongst adults and adolescents, vaccination of CYP under 16 years was not available at the time of this study. This study sought to understand the views of parents/carers regarding vaccination against COVID-19 for CYP with rheumatic and autoinflammatory diseases.

**Description/Method:**

An online survey was developed and shared via social media and direct contacts, targeted at parents of children with rheumatic and autoinflammatory conditions in Canada. Fieldwork took place during May 2021. Consent was provided during enrolment.

**Discussion/Results:**

A total of 157 responses were received (78% female, median age 12). The primary diagnosis for the majority was Juvenile Idiopathic Arthritis (JIA; 39% polyarticular, 15% oligoarticular, 8% enthesitis-related JIA, 6% psoriatic, and 9% systemic). At the time of completing the survey, the majority of CYP had received no vaccination against COVID-19 (83%), although 17% had received one dose; none had received both doses. The majority of parents/carers (55%) would agree for their child to be vaccinated to prevent COVID-19 if the vaccine was approved and available at no cost, with only 10% saying they would not agree, and 18% unsure. Overall, 40% would allow their child to have the vaccine as soon as it was available, with a further 22% who would prefer to wait, and 10% who will allow their child to have the vaccine only when required to. Reasons given by parents choosing not to vaccinate their child against COVID-19 focussed on perceived safety, apparent lack of testing, and alleged potential damage caused by vaccines. In addition, some respondents advised that they had seen anti-vaccine videos on social media targeted at young people, but would still have their child vaccinated when it was available. The majority (92%) cited their doctor or health professionals would be a key source of information when deciding whether to vaccinate their child; however, this varied significantly (p=.0017, chisquare) based on whether they currently would agree to have their child vaccinated, with only 69% of parents who would not vaccinate their child saying their healthcare professional would influence their decision. Around 38% of parents would be influenced by information from their patient organisation. Parents who indicated they would not be vaccinated themselves were less likely to agree for their child to be vaccinated (p<.001, chi-square).

**Key learning points/Conclusion:**

Healthcare professionals play a vital role in supporting, advising and influencing parental decision-making with regards to COVID-19 vaccination amongst CYP with rheumatic and autoinflammatory conditions, particularly amongst parents/carers who are currently undecided. Working collaboratively with patient organisations to deliver simple, clear advice may help reduce vaccine hesitancy.

